# The glucose-lowering effect of low-dose diacerein and its responsiveness metabolic markers in uncontrolled diabetes

**DOI:** 10.1186/s13104-022-05974-9

**Published:** 2022-03-04

**Authors:** Jintanan Jangsiripornpakorn, Sasima Srisuk, Laor Chailurkit, Hataikarn Nimitphong, Sunee Saetung, Boonsong Ongphiphadhanakul

**Affiliations:** 1 Taksin Hospital, Bangkok, Thailand; 2 Bangkok Metropolitan Administration General Hospital, Bangkok, Thailand; 3 Faculty of Medicine Ramathibodi Hospital, Mahidol University, Bangkok, Thailand; 4 Division of Endocrinology and Metabolism, Department of Medicine, Faculty of Medicine Ramathibodi Hospital, Mahidol University, Rama 6th Road, Bangkok, 10400 Thailand

**Keywords:** Diacerein, Type 2 diabetes, Metabolomics, Glycemic control, Inflammation

## Abstract

**Objective:**

Diacerein inhibits the synthesis and activity of pro-inflammatory cytokines, decreases macrophage infiltration in adipose tissue and thus increases insulin sensitivity and signalling. We conducted this study to determine the efficacy of low-dose diacerein in improving glycaemic control in type 2 diabetes mellitus (T2DM) patients with inadequate glycaemic control and to identify the metabolic determinants for such improvement. We randomised 25 T2DM patients with poor glycaemic control, despite being treated with at least three glucose-lowering agents, to receive diacerein 50 mg once-daily (n = 18) or placebo (n = 17) for 12 weeks. Changes in glycated haemoglobin (HbA1c) were evaluated at the 4th and 12th weeks. Metabolic profiling was performed using liquid chromatography electrospray ionisation quadrupole time-of-flight mass spectrometry.

**Results:**

HbA1c levels were significantly reduced from baseline in the diacerein group at 12 weeks (− 0.6%, *p* < 0.05), whereas fasting plasma glucose (FPG) levels were not significantly decreased (− 18.9 mg/dl, *p* = 0.06). Partial least squares-discriminant analysis demonstrated an association between the serum abundance of threo-isocitric acid (ICA) and HbA1c response in the diacerein group. After adjusting for serum high-sensitivity C-reactive protein, ICA was still significantly related to the change in HbA1c.

*Retrospective trial registration* Current Controlled Trials TCTR20200820004, 20 August 2020.

**Supplementary Information:**

The online version contains supplementary material available at 10.1186/s13104-022-05974-9.

## Introduction

The prevalence of type 2 diabetes mellitus (T2DM) is increasing worldwide in conjunction with increased obesity as well as urbanisation. Although it has been well documented that blood glucose control is important in preventing and reducing the progression of diabetic complications, several patients with T2DM are unable to reach the glycaemic target even being treated with several effective anti-diabetic drugs available in clinical practice [1].

Diacerein has been used as an alternative therapy for osteoarthritis [2]. Studies have shown that diacerein inhibited the synthesis and activity of pro-inflammatory cytokines such as TNF-α, IL-6 and especially IL-1β [3]. Randomised controlled trials conducted in subjects with T2DM have demonstrated that diacerein can be beneficial for glycaemic control [4–6], suggesting that it can be used as a complementary therapy for T2DM. However, the commonly used doses of diacerein are often associated with adverse effects, including diarrhoea and liver dysfunction, and there are no data available in randomised trials regarding diacerein treatment in subjects with uncontrolled T2DM receiving other medications. Profiling the metabolites in bio-fluid using the metabolomics approach has been applied in biomarker discovery for both disease biomarkers and biomarkers for pharmacological responses. Therefore, to alleviate the side effects and enhance the efficacy-to-safety ratio of diacerein, we conducted this study using a lower dose of diacerein and investigated the baseline metabolite profile associated with enhanced glycaemic response.

## Main text

### Materials and methods

This randomised, double-blind, placebo-controlled trial was conducted in patients with T2DM who had inadequate glycaemic control despite receiving at least three glucose-lowering agents. The patients were recruited from the endocrine clinic at Ramathibodi Hospital. Written informed consent was obtained from all participants, and the study was approved by Mahidol University Institutional Review Board.

#### Participants

Patients aged 40–65 years who had T2DM for at least 2 years since diagnosis with a glycated haemoglobin (HbA1c) level of 7.5–10% were included in this study. All subjects were receiving stable dosages (duration ≥ 3 months) of at least three glucose-lowering agents including sulphonylureas: glipizide ≥ 20 mg/day, glibenclamide ≥ 10 mg/day, glimepiride ≥ 4 mg/day, gliclazide ≥ 160 mg/day and gliclazide MR ≥ 120 mg/day; biguanides: metformin ≥ 2000 mg/day; thiazolidinediones: pioglitazone ≥ 30 mg/day; dipeptidyl peptidase 4 (DPP4) inhibitors: sitagliptin 100 mg/day, vildagliptin 100 mg/day, linagliptin 5 mg/day and saxagliptin 5 mg/day; and long-acting and intermediate-acting insulin: glargine, detemir, NPH; or premixed insulin. Patients who had chronic inflammatory disease, chronic steroid use, serum creatinine level > 2 mg/dL or eGFR < 60 ml/min/1.73 m^2^/day, aspartate aminotransferase (AST) and alanine aminotransferase (ALT) levels more than three times the upper normal limit and refused to participate in the research were excluded from the study.

#### Procedures

Before randomisation, baseline data were collected from all eligible participants. Blood samples were drawn after an overnight fasting of more than 8 h. Eligible participants were randomly assigned to receive diacerein 50 mg once-daily or placebo tablets once-daily for 12 weeks. All participants received their current medication with unchanged dosages for 12 weeks. Clinical visits were performed at week 0 (baseline), week 4 and week 12. Adherence to treatment regimen was evaluated based on pill counts and structured interviews. Our study adheres to the CONSORT guidelines and a CONSORT checklist was included as a Additional file 1.

#### Outcomes measured

The primary outcome was to compare the change in HbA1c levels from baseline to week 12 among the control and treatment groups. The secondary outcomes were to compare the change in fasting plasma glucose (FPG) levels from baseline to week 12 among among the treatment groups and to determine the factors that may predict the change in HbA1c and FPG levels. The change in HbA1c (∆HbA1c) was calculated using the HbA1c level at week 12 subtracted by the baseline HbA1c level. The change in FPG (∆FPG) was defined by the FPG level at week 12 subtracted by the baseline FPG level.

### Serum metabolomics analysis

An Agilent 1260 high performance liquid chromatography coupled to an Agilent 6540 UHD accurate-mass quadrupole time-of-flight mass spectrometry with *dual Jet Stream electrospray ionization* (Agilent Technologies, Inc., *Santa Clara*, *CA, US*) was used to separate metabolites in the serum samples. The Masshunter Mass Profiler Professional Software B.12.6.1 (Agilent Technologies, USA) was used to perform a non-targeted metabolomics analysis of the extracted features. The accurate masses of features representing significant differences were explored against the METLIN [6] database.

### Statistical analysis

Data with normal distribution are presented as mean ± SD, and those with not normal distribution are presented as median (interquartile range). Student’s *t*-test and the Wilcoxon signed-rank test was used to analyse the differences in continuous variables where appropriate. Multiple regression analysis was used to identify independent associations of relevant variables. A P value < 0.05 was considered to indicate statistical significance.

For the metabolite profile, partial least squares-discriminant analysis (PLS-DA) modelling was performed using the ropls R package. Feature selection was performed using a partial least squares-based algorithm for parsimonious variable selection [7], with the VIP threshold set to 1.5.

### Results

#### Baseline characteristic data

There were 35 patients with T2DM who met the inclusion and exclusion criteria. Patients were randomly assigned to two treatment groups, consisting of 18 patients in the diacerein group and 17 patients in the placebo group. All the 35 patients completed the study at 12 weeks. There was no change or adjustment of glucose-lowering agents during the study period.

Table 1 shows the baseline characteristics of the study subjects. There were no significant differences between the two groups in terms of their baseline characteristics, including age, gender and BMI. The duration of diabetes and serum hsCRP levels were also comparable.

**Table 1:** Clinical characteristics of participants at baseline

Baseline characteristic	Diacerein group (n = 18)	Placebo group (n = 17)	P-value
Age (year)	54.7 ± 1.2	52 ± 3.1.7	0.23
Female sex, n (%)	13 (72.2)	9 (52.9)	0.51
Duration of diabetes (year)	12.5 ± 1.5	11.4 ± 1.1	0.55
Body mass index (kg/m^2^)	29.3 ± 1.0	29.5 ± 1.2	0.86
Fasting plasma glucose (mg/dL)	162.9 ± 8.7	165.9 ± 10.7	0.71
HbA1c (%)	8.4 ± 0.1	8.5 ± 0.2	0.83
hsCRP (mg/dL)*	0.86 (0.62–2.31)	1.42 (1.11–2.22)	0.12

Values are shown as mean ± SE

*HbA1c* glycated haemoglobin, *hsCRP* High Sensitivity C-Reactive Protein

*Median (interquartile range)

#### Change in FPG and HbA1c levels after intervention

Table 2 shows the clinical and laboratory parameters at baseline, week 4 and week 12. There was a significant reduction in HbA1c levels from baseline in the diacerein group (*p* < 0.05), whereas the FPG level appeared to decrease but did not reach statistical significance (*p* = 0.06). There were no significant reductions in FPG and HbA1c levels in the placebo group. Nevertheless, the change in FPG or HbA1C levels after intervention between the two groups was not statistically different (*p* = 0.26 and *p* = 0.55 for FPG and HbA1c, respectively).

**Table 2:** Glycated haemoglobin (HbA1c), fasting plasma glucose (FPG), AST and ALT levels at baseline and at weeks 4 and 12 after treatment

	Diacerein group (n = 18)	Placebo group (n = 17)
HbA1c (%)		
Baseline	8.4 ± 0.1	8.5 ± 0.2
Week 4	8.1 ± 0.2 (*p* = 0.14)	8.5 ± 0.1 (*p* = 0.76)
Week 12	7.8 ± 0.2 (*p* < 0.05)	8.2 ± 0.2 (*p* = 0.06)
FPG (mg/dL)		
Baseline	162.9 ± 8.7	165.9 ± 10.7
Week 4	164.4 ± 9.9 (*p* = 0.88)	164.4 ± 9.9 (*p* = 0.88)
Week 12	144.0 ± 0.9 (*p* = 0.06)	161.9 ± 13.6 (*p* = 0.63)
AST (U/L)		
Baseline	29.3 ± 2.9	36.5 ± 3.4
Week 4	28.8 ± 3.0 (*p* = 0.76)	36.1 ± 3.1 (*p* = 0.90)
Week 12	24.9 ± 2.1 (*p* = 0.08)	37.6 ± 3.4 (*p* = 0.69)
ALT (U/L)		
Baseline	28.4 ± 2.2	28.5 ± 1.5
Week 4	27.9 ± 2.0 (*p* = 0.65)	32.5 ± 3.2 (*p* = 0.18)
Week 12	26.0 ± 1.7 (*p* = 0.11)	31.6 ± 1.7 (*p* = 0.09)

Values are shown as mean ± SE unless otherwise indicated

P-values are versus baseline

#### Factors predicting the changes in HbA1c or fasting plasma glucose levels between baseline and week 12

The hsCRP levels in the diacerein group appeared to decrease after 12 weeks but did not reach statistical significance (median hsCRP = 1.42 and 1.19 mg/dL at 0 and 12 weeks, respectively; *p* = 0.59). A positive correlation was observed between baseline hsCRP levels and the change in HbA1c levels (*r* = 0.50, *p* < 0.01), but not for FPG levels (*r* = 0.09, *p* = 0.61), in all participants. Multiple regression analysis adjusted for age, gender, BMI and the type of treatment revealed that the change in HbA1c level correlated positively with baseline hsCRP level independent of age, gender and baseline BMI. No independent association of hsCRP with the change in FPG level was found (Additional file 1: Table S1).

#### Metabolite profiling

**Fig. 1:**
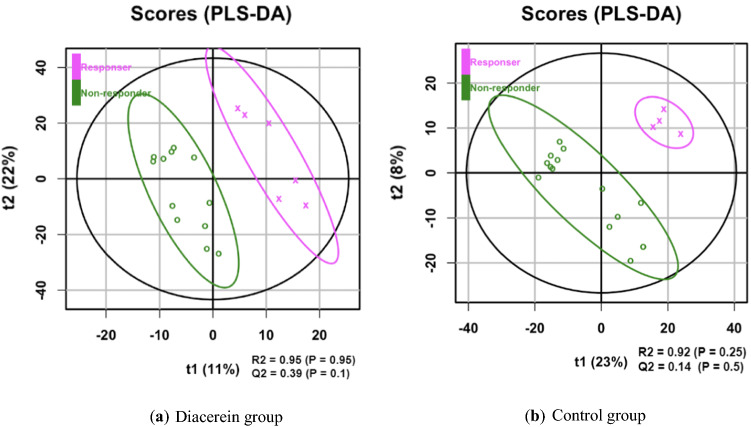
Score plots and the associated R2, Q2 and P values from the permutation test

To identify the metabolites that may be associated with the HbA1c response in the diacerein and control groups, further metabolomics analyses were performed. Subjects were classified into two groups, i.e. those with ≥ 1% decrease in HbA1c levels after 12 weeks and those with a lower degree of response. Metabolites at baseline were analysed using partial least squares-discriminant analysis (PLS-DA) to classify subjects according to the category of HbA1c response. The score plots and the associated R2, Q2 and P values from the permutation test are presented in Fig. 1. Metabolites with top 5 variable importance in projection (VIP) scores are shown in Additional file 1: Table S2.

In the diacerein group, 5-acetylamino-6-formylamino-3-methyluracil (AFMU) and threo-isocitric acid were the two topmost identifiable metabolites associated with response in HbA1c, whereas buprenorphine (BNP) was the top identifiable metabolite associated with a > 1% decrease in HbA1c after 12 weeks in the control group. Considering the change in HbA1c level as the dependent variable and the abundance of AFMU and ICA together with the hsCRP and baseline BMI as independent variables, the analysis showed that ICA was negatively related to the change in HbA1c independent of AFMU, hsCRP and BMI, whereas AFMU was not significantly associated with the change in HbA1c level after adjusting for ICA. In the control group, BNP was not independently associated with the change in HbA1c level after controlling for hsCRP and BMI values (Additional file 1: Table S3).

### Discussion

In the present study, we investigated the efficacy of diacerein in improving glycaemic control in patients with uncontrolled T2DM and observed that even in poorly controlled T2DM, diacerein could reduce HbA1c levels after 12 weeks of treatment. These results are similar to those of recently conducted randomised controlled trials in drug-naive patients with T2DM, which showed that HbA1c levels and FPG levels significantly decreased after diacerein administration [4–6]. However, unlike the results of previous studies, the change in HbA1c and FPG levels was not different from that in the control group in our study. This could be explained by the difference in the characteristics of subjects, small sample sizes, the difference in duration of diabetes and the lower doses of diacerein used in our study.

CRP is a marker of inflammation that is produced in the liver [8]. It is associated with hyperglycaemia and insulin resistance [9]. Previous studies have demonstrated that anti-inflammation treatments in T2DM such as anakinra [10, 11] and gevokizumab [12], decrease CRP levels which correlate with the reduction in HbA1c levels and improvement in insulin secretion [13, 14]. Diacerein might also improve glycaemic control by reducing systemic inflammation. It has been shown in mice that diacerein reduces the expression and activity of pro-inflammatory mediators accompanied by increased insulin sensitivity primarily in the liver and adipose tissue [15]. Consistent with this notion, we found that baseline hsCRP levels significantly positively correlated with the change in HbA1c levels. hsCRP levels are known to vary according to age, race, gender and BMI [16]. After adjusting for age, gender, BMI and the type of treatment intervention, hsCRP remained a significant predictor of the change in HbA1c levels. This implied that baseline hsCRP levels were associated with greater changes in HbA1c, suggesting that increased inflammation could diminish the glucose-lowering effect of any intervention.

In the present study, we found that ICA was associated with the degree of glycaemic response after diacerein treatment. ICA is the substrate of isocitrate dehydrogenase (IDH) that occurs in two structurally distinct forms, i.e. a mitochondrial and a soluble or cytoplasmic form. IDH, particularly the cytoplasmic form, has been shown to be involved in the regulation of insulin secretion from pancreatic beta cells by modulating cytosolic NADPH content [17], and a knockdown of IDH in pancreatic beta cells resulted in an impairment of insulin secretion [18]. The mechanism linking this phenomenon to insulin secretion has been shown to involve an IDH-dependent generation of NADPH and the subsequent glutathione reduction, thereby contributing to the amplification of insulin exocytosis via sentrin/SUMO-specific protease-1 [19]. It is likely that the association between serum ICA and glycaemic response to diacerein found in the present study could reflect such action of IDH on glucose homeostasis.

We also identified AFMU as a potential biomarker for the glycaemic response of diacerein. AFMU is a metabolite of caffeine that results from the acetylation of paraxanthine by N-acetyltransferase [20]. N-acetylation by NAT2 and NAT1 is a major route of biotransformation for xenobiotics containing primary arylamine or a hydralazine group [21]. It is likely that the association between AFMU and response to diacerein found in the present study could be a result of NAT influencing both the metabolism of caffeine and diacerein action, and suggests that NAT activity may partly determine the responsiveness to diacerein.

### Conclusions

Add-on diacerein treatment for 3 months in T2DM patients with inadequate glycaemic control may improve glycaemic control depending on baseline serum ICA.

## Limitations

The limitations of our study include its short duration and small sample size. Further investigations with longer duration and larger sample size are therefore warranted. Moreover, subjects in our study were treated with half the normal doses of diacerein. It is possible that a higher dose of diacerein (100 mg once-daily) might improve the glycaemic outcome associated with diacerein.

## Supplementary Information

**Additional file 1:** **Table S1.** Multivariate analyses showing the independent effect of hsCRP on the change in HbA1c level at 12 weeks. **Table S2.** Metabolites with top 5 variable importance in projection (VIP) scores associated with the response in glycated haemoglobin (HbA1c) to diacerein (a) and control (b) group. **Table S3.** Robust multiple regression analyses of the association between metabolites and changes in glycated haemoglobin (HbA1c) after 12 weeks in the diacerein (a) and control (b) groups.

## Data Availability

The data used to support the findings of this study are available from the corresponding author upon request.

## Abbreviations

T2DM: Type 2 diabetes mellitus
HbA1c: Glycated haemoglobin
ICA: Threo-isocitric acid
DPP4: Dipeptidyl peptidase 4
AST: Aspartate aminotransferase
ALT: Alanine aminotransferase
FPG: Fasting plasma glucose
MFE: Molecular feature extraction
PLS-DA: Partial least squares-discriminant analysis
BMI: Body mass index
VIP: Variable importance in projection
AFMU: 5-Acetylamino-6-formylamino-3-methyluracil
BNP: Buprenorphine
hsCRP: High Sensitivity C-Reactive Protein
IDH: Isocitrate dehydrogenase
