# Supplementary material for: The glucose-lowering effect of low-dose diacerein and its responsiveness metabolic markers in uncontrolled diabetes
Source: BMC Res Notes. 2022 Mar 4;15:91. doi: 10.1186/s13104-022-05974-9 (PMC8896078; doi:10.1186/s13104-022-05974-9)
Supplement: Supplementary file 1 — Additional file 1: Table S1. Multivariate analyses showing the independent effect of hsCRP on the change in HbA1c level at 12 weeks. Table S2. Metabolites with top 5 variable importance in projection (VIP) scores associated with the response in glycated haemoglobin (HbA1c) to diacerein (a) and control (b) group. Table S3. Robust multiple regression analyses of the association between metabolites and changes in glycated haemoglobin (HbA1c) after 12 weeks in the diacerein (a) and control (b) groups. [file 13104_2022_5974_MOESM1_ESM.docx]

**Table S1.** Multivariate analyses showing the independent effect of hsCRP on the change in HbA1c level at 12 weeks.

| **Variables** | **∆ HbA1c** | | **∆ FPG** | |
| --- | --- | --- | --- | --- |
|  | **Regression coefficient (SE)** | **P value** | **Regression coefficient (SE)** | **P value** |
| ln(hsCRP) (mg/dL) | 0.64 ± 0.16 | <0.01 | 7.73 ± 7.93 | 0.32 |
| Age (year) | 0.01 ± 0.02 | 0.71 | −0.12 ± 1.04 | 0.91 |
| Female | −0.41 ± 0.27 | 0.14 | −7.57 ± 13.08 | 0.57 |
| BMI (kg/m^2^) | −0.08 ± 0.03 | <0.05 | −2.71 ± 1.57 | 0.10 |
| Diacerein group | 0.09 ± 0.26 | 0.72 | −4.91 ± 12.67 | 0.70 |

HbA1c **=** glycated haemoglobin, hsCRP **=** High Sensitivity C-Reactive Protein. Ln = Natural logarithm, BMI = body mass index.

**Table S2** Metabolites with top 5 variable importance in projection (VIP) scores associated with the response in glycated haemoglobin (HbA1c) to diacerein (a) and control (b) group.

(a) Diacerein group

| **Metabolites** | **VIP score** |
| --- | --- |
| 5-Acetylamino-6-formylamino-3-methyluracil | 2.56 |
| Threo-isocitric acid | 2.34 |
| C_24_H_35_N_10_O_2_S | 2.33 |
| C_13_H_18_N_5_O_8_ | 2.32 |
| C_44_H_48_N_2_O_10_ | 2.25 |

(b) Control group

| **Metabolites** | **VIP score** |
| --- | --- |
| Buprenorphine | 2.57 |
| C_12_H_27_NO_2_ | 2.36 |
| C_22_H_19_N_2_ | 2.32 |
| C_44_H_48_N_2_O_10_ | 2.12 |
| C_35_H_39_N_30_O_2_S | 2.04 |

**Table S3** Robust multiple regression analyses of the association between metabolites and changes in glycated haemoglobin (HbA1c) after 12 weeks in the diacerein (a) and control (b) groups.

1. Diacerein group

| **Variables** | **Regression coefficient** | **P value** |
| --- | --- | --- |
| ln(hsCRP) (mg/dL) | 0.34 ± 0.19 | 0.09 |
| ICA (abundance) | 9.31 × 10^-7^ ± 2.99 × 10^−7^ | <0.01 |
| AFMU (abundance) | 1.20 × 10 ^−7^ ± 6.88 × 10^−7^ | 0.11 |
| Baseline BMI (kg/m^2^) | 0.03 ± 0.04 | 0.49 |

Ln = Natural logarithm, hsCRP **=** High Sensitivity C-Reactive Protein, ICA = Threo-isocitric acid, AFMU = 5-Acetylamino-6-formylamino-3-methyluracil, BMI = body mass index.

1. Control group

| **Variables** | **Regression coefficient** | **P value** |
| --- | --- | --- |
| ln(hsCRP) mg/dL) | 0.34 ± 0.23 | 0.2 |
| BNP \|(abundance) | −8.80 × 10^−7^± 8.61 × 10^−7^ | 0.33 |
| Baseline BMI (kg/m^2^) | −0.08 ± 0.04 | 0.09 |

Ln = Natural logarithm, hsCRP **=** High Sensitivity C-Reactive Protein, BNP**:** buprenorphine, BMI = body mass index.
